# Evaluating interventions for violence prevention using linked MoJ-DfE data: a feasibility study.

**DOI:** 10.23889/ijpds.v7i3.1877

**Published:** 2022-08-25

**Authors:** Rosie Cornish, Kate Tilling, Iain Brennan

**Affiliations:** 1 University of Bristol; 2 University of Hull

## Objectives

To assess the feasibility of using linked education and offending data (from the National Pupil Database, Department for Education and the Police National Computer, Ministry of Justice) to identify matched control groups to evaluate violence prevention interventions.

## Approach

We simulated a plausible intervention (multi-systemic therapy aimed at high-risk young people living in high-risk areas) aimed at reducing the rate of serious violent offending between the ages of 15 and 18 years. We separately simulated an intervention in London and one outside London. We selected eligible individuals aged 14 years for inclusion in the intervention group, modelled the predictors of serious violent offending. then used two different matching algorithms – prognostic score matching and (coarsened) exact matching – to identify matched controls. We compared their effectiveness by measuring the observed rates of serious violence in the two groups.

## Results

The dataset we used dataset included just under 1.5 million individuals born between 1st September 1995 and 31st August 1998 with complete data. Consistent with previous research, factors associated with the risk of serious violence included deprivation, geographical region, sex, ethnicity, attainment, school absence and exclusion, being in care of the local authority or classified as in need, as well as prior offending and some school-level factors. Exact matching or coarsened exact matching was more successful than prognostic score matching at selecting suitable control groups, both within and outside London. Within London, exact matching on sex, ethnicity and any offending before age 14 gave a suitable control group; outside London it was necessary to match on a few additional characteristics in order to obtain well-balanced groups.

## Conclusion

The linked dataset can feasibly be used to generate suitable matched control groups to evaluate violence prevention interventions; exact matching on key characteristics is potentially the optimal solution. Its utility in practice will depend on regular data updates and having an efficient mechanism for accessing the data for such purposes.

